# Effect of Maltodextrin and Soy Protein Isolate on the Physicochemical and Flow Properties of Button Mushroom Powder

**DOI:** 10.3389/fnut.2022.908570

**Published:** 2022-05-27

**Authors:** Rafeeya Shams, Jagmohan Singh, Kshirod K. Dash, Aamir Hussain Dar, Gulzar Ahmad Nayik, Mohammad Javed Ansari, Hassan A. Hemeg, Abdelhakam Esmaeil Mohamed Ahmed, Ayaz Mukarram Shaikh, Béla Kovács

**Affiliations:** ^1^Department of Food Science and Technology, Sher-e-Kashmir University of Agricultural Sciences and Technology, Jammu, India; ^2^Department of Food Processing Technology, Ghani Khan Choudhury Institute of Engineering and Technology, Maligram, India; ^3^Department of Food Technology, Islamic University of Science and Technology, Awantipora, India; ^4^Department of Food Science and Technology, Government Degree College Shopian, Srinagar, India; ^5^Department of Botany, Hindu College Moradabad, Mahatma Jyotiba Phule Rohilkhand University, Bareilly, India; ^6^Department of Medical Laboratory Technology, College of Applied Medical Sciences, Taibah University, Medina, Saudi Arabia; ^7^Institute of Food Science, University of Debrecen, Debrecen, Hungary; ^8^Faculty of Forestry, University of Khartoum, Khartoum North, Sudan

**Keywords:** maltodextrin, soy protein isolate, freeze-drying, cabinet drying, bioactive compounds, powder efficiency, button mushroom

## Abstract

In this investigation, the effect of different drying techniques, such as freeze-drying and cabinet drying, with two different carrier agents, such as maltodextrin (MD) and soy protein isolate (SPI), at different levels (10, 15, and 20%) on button mushrooms has been revealed. The results showed that the button mushroom powders (BMPs) formulated with SPI as a carrier agent had significantly higher powder yield, hygroscopicity, L^*^, a^*^, and b^*^ values, whereas BMP formulated with MD had significantly higher water activity, solubility index, tapped density, bulk density, and flowability. The highest retention of bioactive compounds was reported in freeze-dried mushroom powder compared to cabinet dried powder using SPI as a carrier agent. Fourier transform infrared (FTIR) analysis confirmed that certain additional peaks were produced in the mushroom button powder-containing SPI (1,035–3,271 cm^−1^) and MD (930–3,220 cm^−1^). Thus, the results revealed that SPI showed promising results for formulating the BMP using the freeze-drying technique.

## Introduction

Mushrooms are edible fungi, and their production and consumption have substantially increased due to their nutritive value, flavor, and high delicacy (1, 2). In India, the mushroom industry is dominated by white button mushrooms, which is a largely sophisticated and capital-intensive operation. According to the latest production data (official data from ICAR-DMR, Solan), the total share of button mushrooms in India is the highest at 73%, followed by oyster mushrooms at 16%. Button mushrooms exhibit a soft texture, are perishable, and deteriorate quickly after harvest (3). Mushrooms are an excellent source of protein, vitamins, minerals, and antioxidants (4) and have potential therapeutic applications, such as the prevention of diabetes, cancer, and cardiac ailments (5), but are highly susceptible to microbial growth due to their higher moisture content ranging from 85 to 92%. The nutritional profile and unique umami flavor of button mushrooms are responsible for their extended cultivation and use. Button mushrooms are highly perishable due to their browning reactions and can open their umbrella with a limited shelf life. Therefore, a large proportion of mushrooms are subjected to drying processes for further use (6). Drying of mushrooms by traditional techniques is commonly used to regulate water activity; however, it results in profound nutrition loss (7). Freeze-drying, a low-temperature process, is a suitable method for drying bioproducts, causing a minimal loss of key functional ingredients (8) compared to thermal processing techniques. Despite being a capital-intensive process, it allows the development of high-quality dried food products. In the freeze-drying process, sublimation is responsible for removing water from the frozen product, which decreases bulk and tapped densities and increases particle porosity (9). Fruit-based powder products possess limitations such as enhanced hygroscopicity and flaking attributed to the availability of sugars and acids with lower molecular weight and to having lower glass transition temperature (10). Therefore, the incorporation of carrier agents such as high molecular weight carbohydrates (maltodextrins (MDs), waxy starch, gum arabic, and microcrystalline cellulose), soy protein isolates (SPIs), and organic solvents imbibes significant potential to produce powders with reduced hygroscopicity, increased powder yield, and improved thermal and microstructural properties (11, 12). Further, carrier agents improve the oxidative stability of the powder. Recently, protein-based carrier agents like SPI have been proven to be highly efficient in producing high product yields, even when used at lower concentrations, as compared to polysaccharides such as MD (13). Each of these carriers shows advantages and disadvantages in terms of cost, process efficiency, and the impact on final powder's properties. One of the disadvantage of using MD under storage conditions at high relative humidity is powder stickiness (14, 15).

Mushrooms are used in noodle products because of their unique flavors and health-promoting characteristics. Incorporating mushrooms into noodles would support the development of key organoleptic features, including the appearance, texture, and flavor of the developed product. Based on this, the objective of this research is to formulate a mushroom-based noodle, and the impact of mushrooms was assessed in terms of their physicochemical characteristics and textural aspects.

## Materials and Methods

### Raw Materials

Fresh button mushrooms were procured from the Division of Plant Pathology, SKUAST-Jammu. Button mushrooms were considered mature when the caps are well-rounded and the partial veil is totally intact. The length-to-thickness ratio of the stipe (stalk) should be small. The length of the drape should be long enough to allow minor trimming without cutting flush with the veil. Button mushroom caps have an average diameter of 4.51 ± 0.87 cm and are linked to short truncated stems with a height of 7.21 ± 1.23 cm. Raw materials were collected, washed, and cut into thin slices followed by drying.

### Drying of Button Mushrooms

#### Freeze-Drying

**Figure 1:**
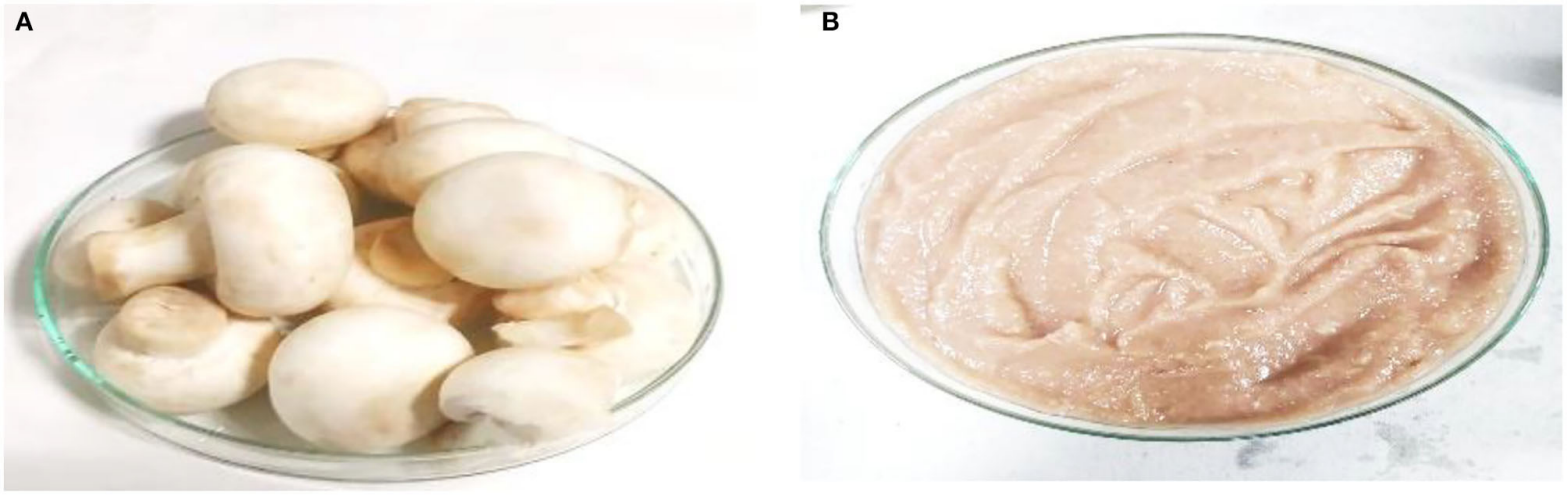
**(A)** Fresh raw button mushroom and **(B)** the paste of button mushroom prior to drying.

**Figure 2:**
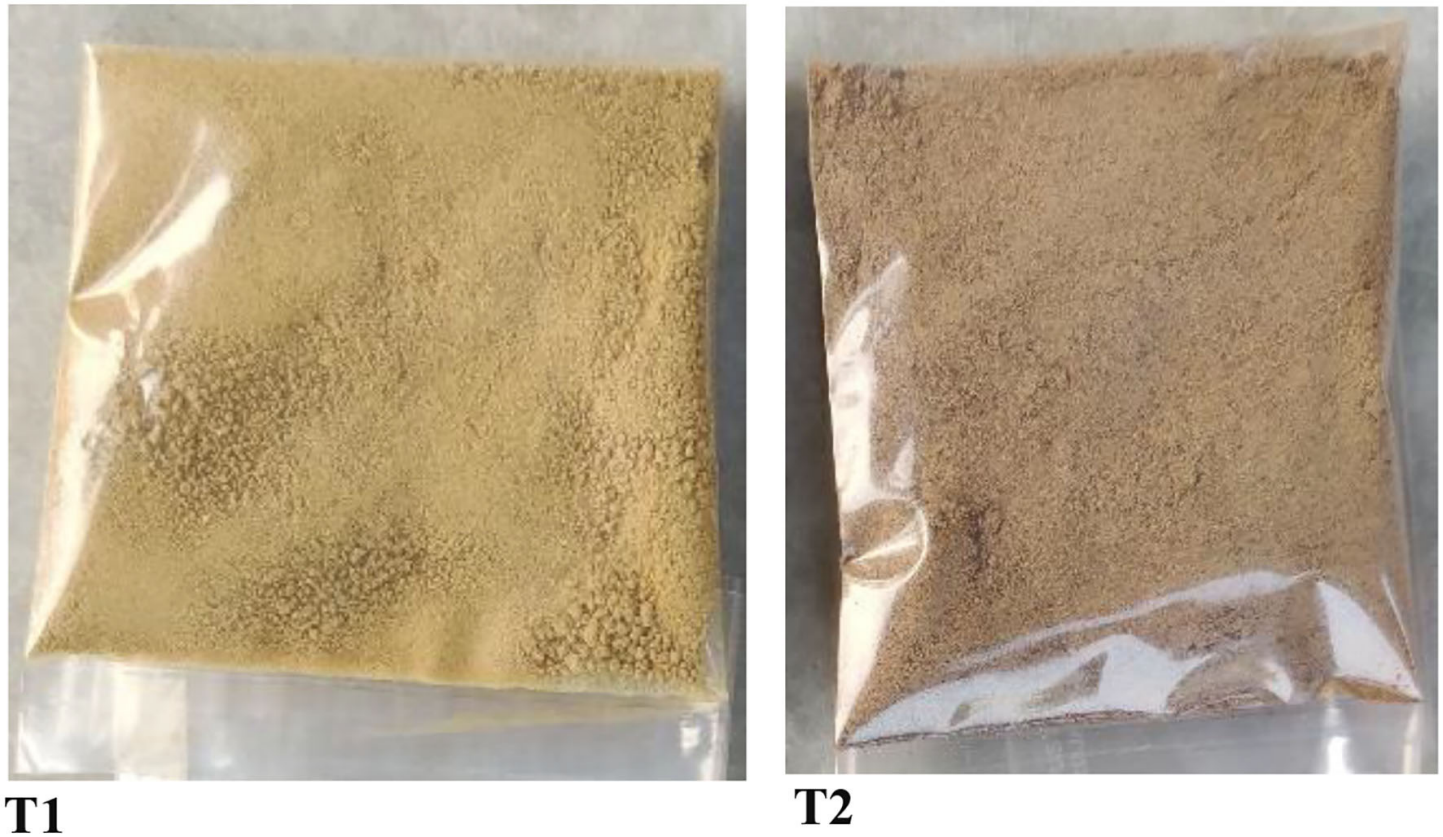
Freeze-dried untreated button mushroom powder (BMP) (T1) and cabinet dried untreated BMP (T2).

**Figure 3:**
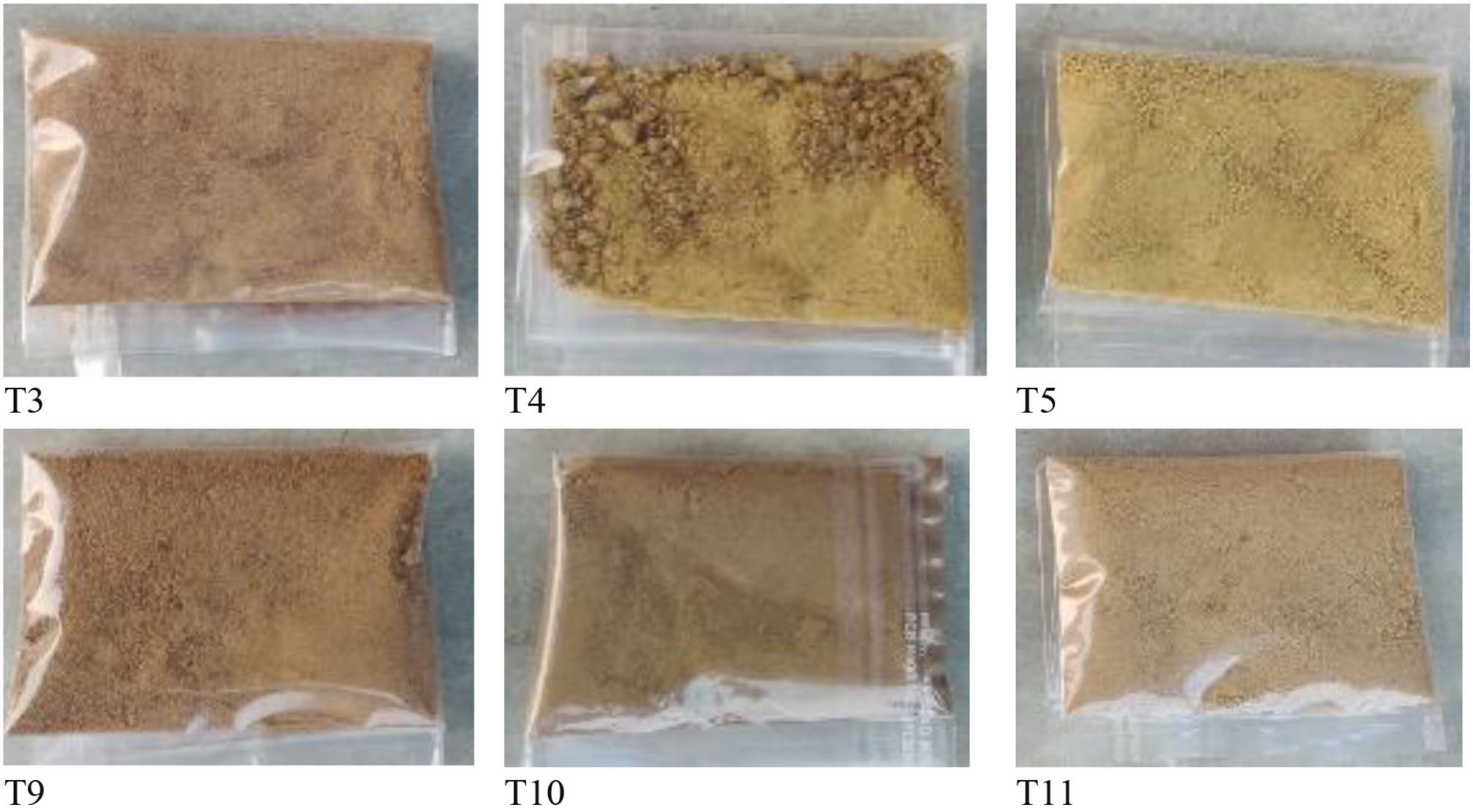
Various forms of pretreated freeze-dried BMP.

**Figure 4:**
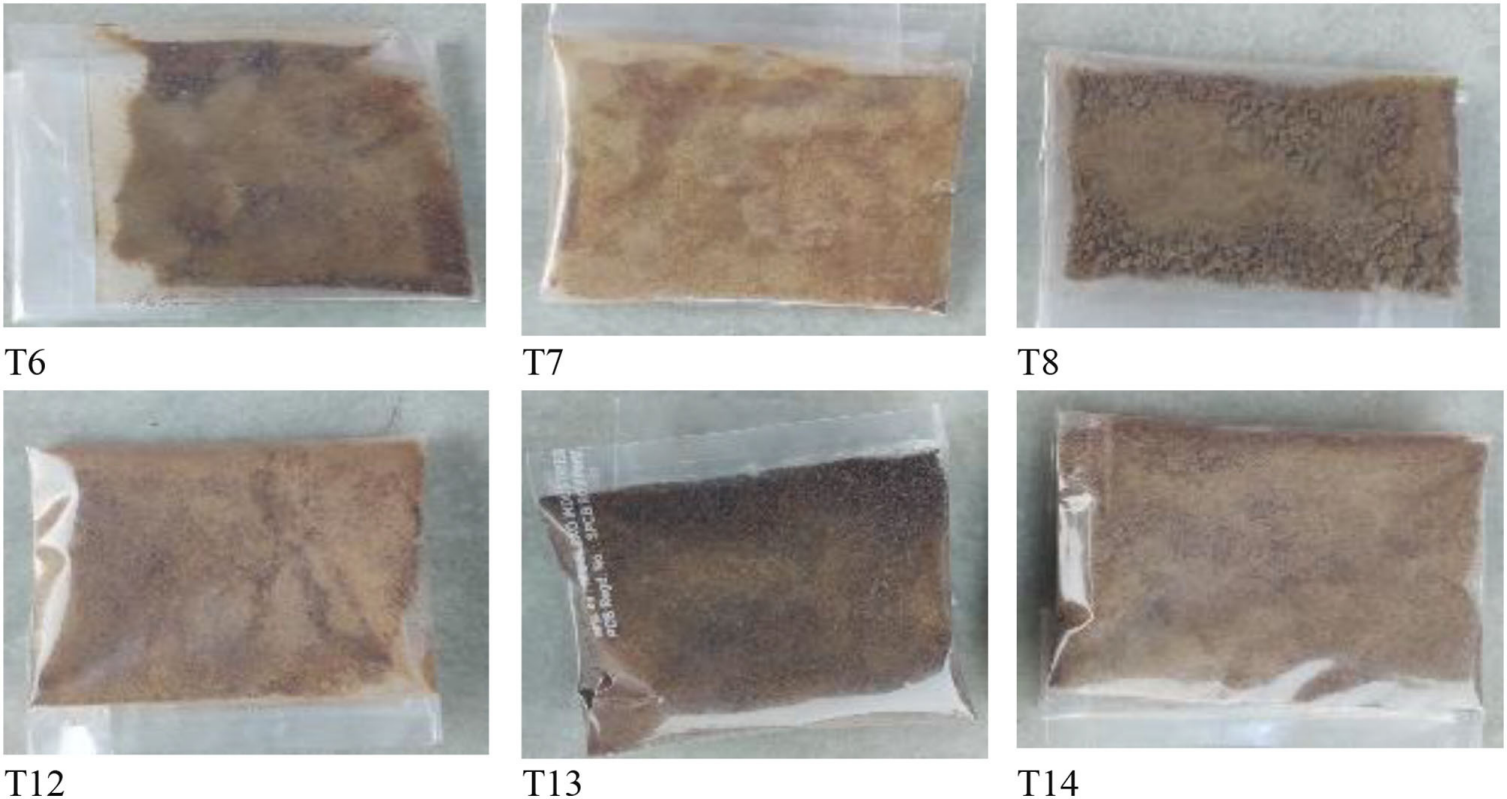
Various forms of pretreated cabinet dried BMP.

Button mushrooms (*Agaricus bisporus*) were selected, washed, and cut into thin slices to provide uniform mixing with carrier agents. The samples were mixed with different carrier agents at different concentrations by adding 25 ml of distilled water to formulate the paste for proper mixing for drying purposes. In freeze-drying, mushroom slices were frozen at a temperature of −80°C in a conventional freezer and then subjected to freeze-drying in a freeze-dryer (SP Scientific VirTis Sentry 2.0, USA) at a pressure of 5 mTorr (0.666 Pa) for a period of 38 h. The obtained flakes were ground using a Mixer Grinder (Philips, India) (as shown in Figures 1–4).

#### Cabinet Drying

In the cabinet drying process, the mushroom samples were washed, cut into thin slices, and dried at 50°C till the constant weight was obtained by grinding using a mixer grinder (Philips, India) in a similar way as in the case of a freeze dryer (as shown in Figures 1–4). The dried mushroom samples were then sealed in plastic pouches and then stored in frozen conditions for further analysis. Sample preparation was carried out using the different formulations (Table 1).

**Table 1:** Ratio of mushroom paste, maltodextrin (MD), and soy protein isolate (SPI) for making the button mushroom powder (BMP).

**Treatment**	**Mushroom paste: Maltodextrin: soy protein isolate**	**Drying method**
T_1_	100:0:0	Freeze-drying
T_2_	100:0:0	Cabinet drying
T_3_	90:10:0	Freeze-drying
T_4_	85:15:0	Freeze-drying
T_5_	80:20:0	Freeze-drying
T_6_	90:10:0	Cabinet drying
T_7_	85:15:0	Cabinet drying
T_8_	80:20:0	Cabinet drying
T_9_	90:0:10	Freeze-drying
T_10_	85:0:15	Freeze-drying
T_11_	80:0:20	Freeze-drying
T_12_	90:0:10	Cabinet drying
T_13_	85:0:15	Cabinet drying
T_14_	80:0:20	Cabinet drying

### Functional Properties

#### Power Yield

Power yield was determined by calculating product recovery as a percentage ratio of the total mass of product recovery to the mass of the extract injected into the system.

#### Water Activity (a_w_)

The sample was filled (3/4th) into a cup of water activity meter (Aqua lab Pre, Decagon Device, USA). The instrument was calibrated as per the instruction manual calibration. The sample was kept in a cup until a constant reading was obtained.

#### Hygroscopicity

Hygroscopicity was calculated by weighing 1.5 g of the powder in an airtight container in a saturated solution of Na_2_CO_3_, and then, weighing was carried out after 1 week. Hygroscopicity was calculated as 1 g of adsorbed moisture per 100 g of the sample (16).

#### Solubility Index

The solubility index was measured by the method given by Hogekamp and Schubert (17). A 5-g sample was thoroughly mixed with 50 ml of distilled water. The mixture was then placed in a centrifuge tube and incubated in a water bath at 37°C for 30 min followed by centrifugation at 1,140 rpm for 20 m (Model-Remi PR-24). Finally, the supernatant was removed, collected in a pre-weighed Petri plate, and dried at 105°C for 4 h. The solubility index (%) was calculated using Equation (1):


(1) $$\begin{array}{l} \text{Solubility index }\left(\%\right)=\frac{\text{Weight of the dried supernatant }\left(\text{g}\right)}{\text{Weight of the sample }\left(\text{g}\right)}\\ \;\times \;100 \end{array}$$

### Bulk Density, Tapped Density, Flowability (Carr Index), and Cohesiveness (Hausner Ratio)

The bulk density (ρ_*B*_, g/ml) and tapped density (ρ_*T*_, g/ml) of the mushroom powder were determined using the methods given by Santhalakshmy et al. (18) and Ozdikicierler et al. (19), as shown in Equations (2) and (3), respectively. The flowability of the sample was measured using the Carr index (CI), and the cohesiveness was measured using the Hausner ratio (HR), as described by Jinapong et al. (20) and shown in Equations (4) and (5), respectively:


(2) $$\begin{array}{r} Bulk\;density,\;\rho_{B}=\;\frac{W}{V}, \end{array}$$

(3) $$\begin{array}{r} Tapped\;density,\;\rho_{T}=\;\frac{M}{V_{t}}, \end{array}$$

(4) $$\begin{array}{r} Carr\;index,\;CI\;\left(\%\right)=\;\frac{\rho_{B}-\;\rho_{T}}{\rho_{T}}, \end{array}$$

(5) $$\begin{array}{r} HR=\;\frac{\rho T}{\rho B}. \end{array}$$

### Hunter Color Value

The color parameter of the mushroom dried by different methods was measured with a Hunter Lab color analyzer (Hunter Lab D-25, Ruston, USA) and expressed in terms of L^*^, a^*^, and b^*^ values. The parameter L^*^ indicated lightness or whiteness, a^*^ indicated redness or greenness, and b^*^ indicated yellowness or blueness. The instrument was first calibrated using a standard black tile and then calibrated using a standard white tile. The L^*^, a^*^, and b^*^ values were obtained by placing a sample handling dish filled with dried powder in the analyzing port. On the other hand, the whiteness index (WI) of samples was calculated using the formula given in Equations (6) and (7):


(6) $$\begin{array}{r} WI=100-\;\sqrt{\left(100-\;L^{*}\right)2\;+\;a^{*}2\;+\;b^{*}2}, \end{array}$$

(7) $$\begin{array}{r} \Delta E^{*}=\;\sqrt{\left(L^{*}c-\;L^{*}s\right)2\;+\;\left(a^{*}c\;a^{*}s\right)2\;+\;\left(b^{*}c\;b^{*}s\right)2}. \end{array}$$

### Bioactive Properties

#### Estimation of Total Phenolic Content

The total phenolic content (TPC) in the mushroom powder was calculated using the spectrophotometric method given by Sawczuk et al. (21). The Folin–Ciocalteu (FC) method was used to determine the TPC of mushroom noodles. About 100 μl of extract solutions of various strengths are added to 1.5 ml of the FC reagent (10%). Approximately 1.5 ml of sodium carbonate (6%) was added to the liquid after 5 min. The measurement was obtained at 725 nm after allowing the mixture to react for 90 min at room temperature. Gallic acid (0.03–0.3 mg/ml) was used to formulate the calibration curve, from which the total polyphenolic content in the extract was determined. The results were expressed as milligrams of gallic acid equivalents per gram of the extract (mg/g).

#### Estimation of Total Flavonoid Content

The total flavonoid content (TFC) in the mushroom powder was evaluated using the procedure described by Ravanfar et al. (22). To determine the TFC, 0.50 ml of each extract stock solution (1 mg ml^−1^) and each dilution of the standard rutin solution (10–100 g ml^−1^) were used in test tubes. Each test tube was filled with 1.50 ml of methanol, 0.10 ml of aluminum chloride solution, 0.10 ml of potassium acetate solution, and 2.80 ml of distilled water and then shaken. Sample blanks for all extracts and standard rutin dilutions were produced in the same method but with distilled water rather than the aluminum chloride solution. All generated solutions were filtered using Whatmann filter paper No. 1 before being measured for absorbance. The absorbance was measured at 510 nm against a suitable blank. A rutin calibration curve was used to determine the TFC. The results were represented as milligrams of rutin equivalents (RE)/g of the extract.

#### Estimation of 2,2-Diphenyl-1-Picrylhydrazyl Radical Scavenging Activity

The free radical scavenging capacity of the sample was calculated with 2,2-diphenyl-1-picrylhydrazyl (DPPH) and DPPH radical scavenging activity using the procedure given by Kiselova-Kaneva et al. (23) and was evaluated using Equation (8):


(8) $$\begin{array}{r} \%\;DPPH\;radical\;scavenging\;=\frac{A_{C}\;A}{A_{C}}\;\times \;100, \end{array}$$

where *A*_*c*_ = absorbance of the control and *A* = absorbance of the sample at 517 nm. The graph plotting each concentration and percentage inhibition yielded the IC_50_ value (the concentration of the sample extract required to inhibit 50% DPPH activity).

#### Reducing Power Activity Assay

The reducing power assay is used to determine the ability of an extract to reduce Fe^3+^ to Fe^2+^. The reducing power of the sample was calculated using the procedure given by Oyaizu (24). The reducing power of the sample extract was measured in terms of EC_50_ (concentration exhibiting 50% absorbance) and compared to that of butylated hydroxytoluene (BHT) and ascorbic acid.

### Fourier Transform Infrared Analysis

Infrared (IR) spectra were measured using a Nicolet 360 Fourier transform IR (FTIR) spectrometer (USA) by mixing 0.1 g of the powdered sample with 0.5 g of potassium bromide for pellet preparation to attain the IR spectrum. The IR region was observed between the range of 650 and 4,000 cm^−1^, representing an average of 32 scans. All spectrums were measured under ambient conditions.

### Statistical Analysis

All samples were prepared at various carrier agent concentrations and analyzed three times. The results were presented in the form of means and standard deviations (SDs). Significant differences between samples were analyzed by *post-hoc* analysis of variance (ANOVA). All statistical analyses were conducted using SPSS software.

## Results and Discussion

### Functional Properties of Button Mushroom Powder

#### Powder Yield

Process yield (powder yield) is an important parameter of the drying process because it influences cost and efficiency. It accounts for the total mass losses, including the extraction and drying processes. However, at a higher dosage of SPI, the moisture content of the button mushroom powder (BMP) also showed a significant increase, which may be due to the higher water holding capacity of proteins than polysaccharides, i.e., MD (25). The data in Table 2 show the effect of treatments on powder yield, revealing that different carrier agents have a significant impact on powder yield. BMP treated with MD showed a significantly lower yield than SPI. The powder yield of BMP varied from 15.90 to 19.90% for MD-treated powder and from 21.00 to 62.10% for SPI-treated powder in both cabinet dried and freeze-dried BMPs, respectively, which was lower than the yield of untreated powder at 15.06 and 18.00% for cabinet dried and freeze-dried powders, respectively. The results depicted that powder recovery increased significantly as the concentration of both carrier agents increased; however, the highest treatment yield was observed for SPI than for MD in freeze-drying. The highest yield in SPI was primarily due to the preferential transfer of more proteins to the interface of the feed solution and the formation of glassy skins (formation of protein-rich films) when exposed to dry and hot air, thus overcoming gumminess and stickiness and bonding (26). Cynthia et al. (27) observed the same trend of incorporation of a carrier agent into the powder yield of tamarind pulp extracts.

**Table 2:** Functional properties of BMP.

**Treatment**	**Mushroom: MD:SPI**	**Drying method**	**Powder yield (%)**	**Water activity (a_**w**_)**	**Hygroscopicity (%)**	**Solubility index (%)**
T_1_	100:0:0	Freeze	18.000 ± 0.469	0.530 ± 0.018	11.180 ± 0.266	80.100 ± 1.256
T_2_	100:0:0	Cabinet	15.060 ± 0.427	0.670 ± 0.014	9.823 ± 0.304	78.800 ± 0.856
T_3_	90:10:0	Freeze	18.860 ± 0.439	0.390 ± 0.013	8.276 ± 0.322	90.000 ± 1.175
T_4_	85:15:0	Freeze	19.520 ± 0.368	0.380 ± 0.024	7.884 ± 0.223	92.500 ± 1.069
T_5_	80:20:0	Freeze	19.890 ± 0.416	0.360 ± 0.012	6.821 ± 0.282	96.000 ± 0.969
T_6_	90:10:0	Cabinet	15.900 ± 0.941	0.620 ± 0.017	9.471 ± 0.392	81.700 ± 0.800
T_7_	85:15:0	Cabinet	16.500 ± 0.502	0.590 ± 0.018	8.931 ± 0.424	83.400 ± 1.194
T_8_	80:20:0	Cabinet	17.500 ± 0.574	0.560 ± 0.023	8.515 ± 0.205	87.600 ± 1.075
T_9_	90:0:10	Freeze	50.300 ± 0.608	0.450 ± 0.011	8.009 ± 0.117	89.300 ± 1.275
T_10_	85:0:15	Freeze	55.800 ± 0.716	0.410 ± 0.007	7.273 ± 0.363	91.000 ± 0.706
T_11_	80:0:20	Freeze	62.100 ± 0.761	0.370 ± 0.009	6.371 ± 0.388	93.200 ± 0.987
T_12_	90:0:10	Cabinet	21.000 ± 0.394	0.580 ± 0.005	9.817 ± 0.232	84.200 ± 0.681
T_13_	85:0:15	Cabinet	21.800 ± 0.439	0.550 ± 0.020	9.038 ± 0.246	86.600 ± 0.612
T_14_	80:0:20	Cabinet	22.000 ± 0.495	0.540 ± 0.016	8.448 ± 0.379	88.300 ± 0.731

*MD, maltodextrin; SPI, soy protein isolate*.

#### Water Activity

The basic preservation principle in drying is to reduce water activity to the level where biochemical reactions and microbial growth are no longer supported. The data in Table 2 show that the water activity of both cabinet dried and freeze-dried BMPs were significantly reduced with the incorporation of carrier agents. The water activity (*a*_*w*_) of the samples decreased from 0.62 to 0.36 for MD-treated powder and from 0.58 to 0.37 for SPI-treated powder in both cabinet dried and freeze-dried BMPs, which was less than that of untreated powder, which had 0.67 and 0.53 in cabinet dried and freeze-dried powders, respectively. The drying technique also significantly influenced the water activity of the samples. Water activity may directly affect the shelf life of the powder. The results depicted that the BMP prepared using MD as a carrier agent possessed significantly lower water activity than the powder produced using SPI. The variation in water activity may be attributed to the high hydrophilic attraction of SPI as compared to MD. The water activity value of 0.20–0.40 for dried foods is regarded as microbiologically safe, increasing the shelf stability of the products (28). Higher water activity may hamper the shelf stability of the products (29). Most bacteria, yeasts, and molds cannot grow below water activity values of 0.87, 0.88, and 0.80, respectively, as a result of decreased water activity (30). However, with a further decrease in water activity, powders produced with carrier agents confer a stable environment due to lower water activity as it is a key factor in maintaining the chemical reaction or chemical stability of the samples (31). However, freeze-dried powders with water activity <0.3 were comparatively less sensitive to microbial damage and more stable for bioactive compounds (32). Gurak et al. (33) observed the same trend, with a water activity of 0.43 for grape juice treated with MD by freeze-drying.

#### Hygroscopicity

Hygroscopicity can affect the powder's storage stability. With the incorporation of carrier agents, the hygroscopicity decreased. The data in Table 2 depict that the hygroscopicity of BMP decreased from 9.5 to 6.8% for MD-treated powder and from 9.8 to 6.4% in SPI-treated powder in both cabinet dried and freeze-dried BMPs, respectively, which was lower than that of untreated powder having hygroscopicity values of 9.8 and 11.2% for cabinet dried and freeze-dried powders, respectively. It was observed that freeze-dried powders had lower values than cabinet dried powders. The hygroscopicity of the powder can explain the water adsorption mechanism in the powders as being attributed to the presence of hydrophilic groups in the structure of each carrier agent. Differences in the values of hygroscopicity might be attributed to the rate at which they absorb water molecules from the environment and the nature of the powder (34). The large particle size, due to increased viscosity of the feed material, can reduce the total surface area, resulting in decreased hygroscopicity (10). The same trend was observed by Etzbach et al. (35) in carotenoid-rich goldenberry, where the hygroscopicity decreased from 17.2 to 14.1%.

#### Solubility Index

Solubility is an essential functional parameter of edible powders, which affects the powder behavior when reconstituted in water (36). The data presented in Table 2 depict that the addition of carrier agents had a significant effect on the solubility. The solubility index increased from 81.7 to 96.0% for MD-treated and from 84.2 to 93.2% for SPI-treated powders in both cabinet and freeze-dried BMPs, than that of untreated powder having solubility index of 78.8 and 80.1% for the cabinet and freeze-dried powders, respectively. Solubility is the key factor in determining the dispersibility and wettability of powders in water (37). Powder solubility may depend on the presence of hydrophilic groups (10). Powder solubility is an essential functional property that has a significant effect on other functional properties such as gelation, emulsification, and foaming properties (38). Zhang et al. (39) also reported that powders prepared with only 20% MD depicted higher solubility due to the high polarity of the polysaccharide. The same trend was also reported by Pudziuvelyte et al. (40) who revealed the solubility of freeze-dried powders in the range of 42.50–92.50%. Grabowski et al. (41) also observed that the solubility of sweet potato powder increased with increasing MD concentration. Freeze-dried powders showed a higher solubility index than cabinet dried powders. It has been also reported that solubility and bulk density are inversely proportional and that protein–protein interaction may increase solubility (15).

### Flow Properties of BMP

#### Bulk Density

Bulk density is defined as the material density when packed or stacked in bulk. Bulk density is commonly used to characterize the end product obtained by drying or milling (42). The bulk density of the dried BMP is essential for determining the powder qualities and industrial operations associated with BMP production, packaging, storage, and distribution (43). The data presented in Table 3 show that the incorporation of carrier agents decreased the bulk density of BMP. The bulk density decreased from 0.510 to 0.400 g/ml for MD-treated powder and from 0.510 to 0.370 g/ml for SPI-treated powder for both cabinet dried and freeze-dried BMPs, respectively, which was less than that of the untreated powder having 0.526 and 0.405 g/ml for the cabinet dried and freeze-dried powders, respectively. The decrease in bulk density with the incorporation of carrier agents might be attributed to the increase in feed viscosity, which resulted in an increase in particle size (44). The rise in feed viscosity with increased protein percentage in the feed material, leading to the creation of aggregates and thus a reduction in bulk density, could be the reason for the decrease in bulk density with the increase in the addition rate of SPI compared to MD (24). Spray-dried bottle gourd and tamarind showed a similar pattern, with bulk density values in the range of 0.36–0.48 and 0.391–0.685 g/ml, respectively (10) (44).

**Table 3:** Flow properties of BMP.

**Treatment**	**Mushroom: MD:SPI**	**Drying method**	**Bulk density (g/ml)**	**Tapped density (g/ml)**	**Carr index (%)**	**Hausner Ratio**
T_1_	100:0:0	Freeze	0.405 ± 0.006	0.490 ± 0.013	17.340 ± 0.227	1.200 ± 0.031
T_2_	100:0:0	Cabinet	0.526 ± 0.005	0.680 ± 0.010	22.640 ± 0.328	1.320 ± 0.024
T_3_	90:10:0	Freeze	0.480 ± 0.013	0.560 ± 0.004	14.280 ± 0.263	1.180 ± 0.053
T_4_	85:15:0	Freeze	0.410 ± 0.012	0.460 ± 0.003	10.860 ± 0.313	1.090 ± 0.036
T_5_	80:20:0	Freeze	0.400 ± 0.013	0.430 ± 0.007	6.970 ± 0.273	1.080 ± 0.041
T_6_	90:10:0	Cabinet	0.510 ± 0.025	0.650 ± 0.007	21.530 ± 0.328	1.280 ± 0.075
T_7_	85:15:0	Cabinet	0.500 ± 0.004	0.600 ± 0.014	16.660 ± 0.380	1.210 ± 0.062
T_8_	80:20:0	Cabinet	0.490 ± 0.009	0.580 ± 0.003	15.510 ± 0.245	1.200 ± 0.035
T_9_	90:0:10	Freeze	0.470 ± 0.010	0.540 ± 0.005	12.960 ± 0.343	1.160 ± 0.017
T_10_	85:0:15	Freeze	0.410 ± 0.015	0.450 ± 0.008	8.800 ± 0.363	1.100 ± 0.028
T_11_	80:0:20	Freeze	0.370 ± 0.015	0.400 ± 0.004	5.000 ± 0.345	1.070 ± 0.009
T_12_	90:0:10	Cabinet	0.510 ± 0.009	0.630 ± 0.008	19.040 ± 0.387	1.250 ± 0.071
T_13_	85:0:15	Cabinet	0.490 ± 0.023	0.540 ± 0.006	9.228 ± 0.467	1.110 ± 0.043
T_14_	80:0:20	Cabinet	0.450 ± 0.021	0.488 ± 0.013	7.780 ± 0.420	1.085 ± 0.027

*MD, Maltodextrin; SPI, soy protein isolate*.

#### Tapped Density

The tapped density of the BMP was based on the true solid density; however, it did not take into account the spaces between the particles (45). Table 3 demonstrates that the tapped density of cabinet dried and freeze-dried BMPs range from 0.43 to 0.65 g/ml for MD-incorporated powder and from 0.40 to 0.63 g/ml for SPI-incorporated powder. The results were lower than those for powders produced without MD and SPI, which had 0.68 and 0.49 g/ml for cabinet dried and freeze-dried BMPs, respectively. The decrease in tapped density with the incorporation of carrier agents indicates an increase in particle size. Low moisture content (reduced water activity) played an essential role in reducing the bulk and tapped density. The increase in the carrier agent concentration provides a large number of ramifications with hydrophilic groups, leading to lower residual moisture content and resulting in low bulk density (10). Bhat et al. (10) also reported reduction in the tapped density from 1.20 to 1.37 g/ml with the incorporation of carrier agents. The same pattern was observed by Sarabandi et al. (46), stating that, as the protein ratio increases, the viscosity of the feed increases, causing a decrease in density and an increase in particle size.

#### Flowability (Carr Index) and Cohesiveness (HR)

The Carr Index (CI) and HR are important in giving information regarding the flowability of the powder (47). Flowability and cohesiveness are the essential handling characteristics of dry powder. The flowability of the powder can be expressed as the Carr index (CI), while the cohesiveness is expressed as the HR (48). The Carr index (CI) and HR for better flowability of powders must be <15 and 1.25%, respectively. Table 3, pertaining to the HR and CI of both freeze-dried and cabinet dried BMPs, shows that, with the incorporation of carrier agents, CI decreased significantly from 21.53 to 6.97% in MD-treated powder and from 19.04 to 5.00% in SPI-treated powder in both cabinet dried and freeze-dried BMPs, respectively, which was less than that of the untreated powder having 22.64 and 17.34% for cabinet dried and freeze-dried BMPs, respectively. HR also decreased with the addition of carrier agents from 1.28 to 1.08 in MD-treated powder and from 1.25 to 1.07 in SPI-treated powder in both cabinet dried and freeze-dried BMPs, respectively, which was less than that of the untreated powder having 1.32 and 1.20 for cabinet dried and freeze-dried powders, respectively. It is evident from Table 3 that the freeze-dried powder will flow better than the powder after cabinet drying. After freeze-drying, the protein particle size increased, and the higher the particle size, the smaller the surface area per unit mass. The lower the possibility of surface interaction, the lower the cohesion, and the greater the flowability (49). HR and CI are highly sensitive to differences in shape and particle size distribution (10). Powder particle flowability was mostly influenced by surface characteristics (globular and smooth, or rough surface with dents) and particle size distribution (50). Tontul et al. (48) also revealed a similar pattern in tomato powder samples.

### Color

Color is an essential quality parameter reflecting the sensory attractiveness and the quality of the powder (51). In dried foods, color is a quality indicator that provides information on the relative color change of fresh and dried materials (52). The incorporation of carrier agents significantly affected the color values (L^*^, a^*^, and b^*^). The data presented in Table 4 show that, as the level of carrier agents increased, the L^*^ value increased, which can be due to the inherent whitish color of the carrier agent, while a^*^ and b^*^ decreased. The L^*^ value increased in both MD- (65.16–85.26) and SPI-treated powders (63.87–76.23) in both cabinet dried and freeze-dried BMPs, which was higher than that of the untreated powder having 60.32 and 73.39 for the cabinet dried and freeze-dried powders, respectively. The addition of different carrier agents significantly reduced a^*^ and b^*^ in both drying procedures. The a^*^ value decreased in both MD- (3.12–0.66) and SPI-treated powders (3.96–0.73) in both cabinet dried and freeze-dried BMPs, respectively, which were less than that of the untreated powder having 3.04 and 0.89 for the cabinet dried and freeze-dried powders, respectively. The b^*^ value decreased in both MD- (11.37–7.87) and SPI-treated powders (12.01–10.37) in both cabinet dried and freeze-dried BMPs, respectively, which was less than that of the untreated powder having 12.26 and 10.22 for the cabinet dried and freeze-dried powders, respectively. Meanwhile, the WI value increased in both MD- (63.21–83.27) and SPI-treated (61.70–74.05) powders in both cabinet dried and freeze-dried BMPs, respectively, which was less than that of the untreated powder having 58.35 and 71.48 for the cabinet dried and freeze-dried powders, respectively. The changes in color parameters largely depend on the type and concentration of carrier agents in the feed solutions, which can be related to polyphenolic changes during drying (53). Ahmed et al. (54) showed that, in purple sweet potato powder, the L^*^ value increased with the incorporation of MD as compared with untreated samples. The color values were attributed to the formation of polymeric anthocyanins. Padzil et al. (28) also observed that the L^*^ value of the treated purple sweet potato extract increased with the incorporation of MD as the MD initially has a white color (L^*^ = 98.18 ± 0.15), whereas the redness (+a^*^) of the treated purple sweet potato extract was significantly decreased as the concentration of MD increased. The increase in lightness and a reduction in chroma (color saturation) could be attributed to the dilution effect (55). The same trend was obtained for powdered pink guava with MD (56) and tamarind pulp with MD–SPI (57). Rocha-Parra also observed a decrease in a^*^ (redness) and an increase in L^*^ (lightness) with the incorporation of carrier agents (58). Bhusari et al. also observed similar results stating that tamarind pulp powder (TPP) with protein concentrate at all levels showed lower values of lightness (L) as compared to TPPs with MD (44). This may be due to the inherent color of the protein concentrate. Also, the a^*^ and b^*^ values decreased as the concentration of carrier agents increased. TPPs with protein concentrate showed high b^*^ values than TPPs with MD (44).

**Table 4:** Color parameters of BMP.

**Treatment**	**Mushroom: MD:SPI**	**Drying method**	L*	a*	b*	**WI**
T_1_	100:0:0	Freeze	73.390 ± 0.273	0.890 ± 0.023	10.220 ± 0.262	71.481 ± 0.125
T_2_	100:0:0	Cabinet	60.320 ± 0.310	3.040 ± 0.052	12.260 ± 0.328	58.358 ± 0.241
T_3_	90:10:0	Freeze	73.050 ± 0.328	2.380 ± 0.038	12.010 ± 0.313	70.399 ± 0.263
T_4_	85:15:0	Freeze	80.690 ± 0.440	1.770 ± 0.085	10.220 ± 0.228	78.081 ± 0.316
T_5_	80:20:0	Freeze	85.260 ± 0.512	0.660 ± 0.018	7.870 ± 0.268	83.278 ± 0.241
T_6_	90:10:0	Cabinet	65.160 ± 0.453	3.120 ± 0.048	11.370 ± 0.440	63.219 ± 0.462
T_7_	85:15:0	Cabinet	71.840 ± 0.335	3.010 ± 0.133	9.380 ± 0.357	70.167 ± 0.329
T_8_	80:20:0	Cabinet	72.680 ± 0.455	2.800 ± 0.153	9.120 ± 0.395	71.062 ± 0.401
T_9_	90:0:10	Freeze	69.040 ± 0.362	2.600 ± 0.143	11.780 ± 0.270	66.773 ± 0.208
T_10_	85:0:15	Freeze	70.440 ± 0.422	1.810 ± 0.108	11.120 ± 0.330	68.366 ± 0.316
T_11_	80:0:20	Freeze	76.230 ± 0.502	0.730 ± 0.020	10.370 ± 0.410	74.056 ± 0.430
T_12_	90:0:10	Cabinet	63.870 ± 0.293	3.960 ± 0.093	12.050 ± 0.170	61.708 ± 0.204
T_13_	85:0:15	Cabinet	69.830 ± 0.382	3.610 ± 0.020	11.810 ± 0.475	67.400 ± 0.375
T_14_	80:0:20	Cabinet	72.110 ± 0.475	3.300 ± 0.160	11.420 ± 0.278	69.682 ± 0.286

*MD, maltodextrin; SPI, soy protein isolate*.

### Bioactive Properties of BMP

#### Total Phenolic Content

Determination of the TPC of carrier agent-treated powder was needed to calculate the powder efficiency. Different concentrations of carrier agents had a significant impact on the phenolic content. The data in Table 5 show that, with an increase in the concentration of carrier agents, the phenolic content decreased from 15.81 to 12.84 mg GAE/g in MD-treated powder and from 15.96 to 12.89 mg GAE/g in SPI-treated powder in both freeze-dried and cabinet dried BMPs, respectively, which was lower than that of the untreated powder having 17.06 and 20.30 mg GAE/g for the cabinet dried and freeze-dried powders, respectively. The powder produced with SPI depicted high retention, while the use of MD caused low TPC retention. This can be attributed to the strong interactions between polyphenols–proteins as compared to polyphenols–carbohydrates (10). TPC methodology is used to determine the number of phenolic compounds based on the formation of molybdate- and phosphotungstate-containing complexes and the electron transfer shown as a blue color in the solution (59). Wang et al. also observed that the TPC of mulberry juice powders was significantly decreased with an increased concentration of MD (49). A similar tendency was found for protein concentrate; TPC decreased significantly with an increase in the total solid content of proteins in the feed solution (60). Leyva-Porras et al. also showed a significant decrease in TPC with the incorporation of MD strawberry juice powder. Boonchu and Utama-ang also found that the TPC decreased in red grape pomace with an increase in MD concentration (59). Muzaffar and Kumar also found similar results stating that an increase in SPI concentration significantly reduced the DPPH radical scavenging activity of powders from 61.91 to 45.81 mg GAE/g, which could be correlated with the concentration effect of SPI at each concentration level (61). Mishra et al. also found a reduction in TPC as the concentration of carrier agents increased. It has been proven that increasing MD concentration in amla juice decreased the TPC, which further led to the decrease in radical scavenging capacity of the prepared powder (62). The overall decline in TPC can be due to enzymatic and oxidative degradation of polyphenols to subunits.

**Table 5:** Bioactive properties of BMP.

**Treatment**	**Mushroom: MD:SPI**	**Drying method**	**TPC (mgGAE/g)**	**TFC (mg RE/g)**	**DPPH radical scavenging activity (IC_**50**_; mg/ml)**	**Reducing power activity (EC_**50**_; mg/ml)**
T_1_	100:0:0	Freeze	20.300 ± 0.502	17.310 ± 0.253	4.060 ± 0.002	0.830 ± 0.105
T_2_	100:0:0	Cabinet	17.060 ± 0.343	16.080 ± 0.078	3.980 ± 0.013	0.760 ± 0.027
T_3_	90:10:0	Freeze	15.810 ± 0.385	13.740 ± 0.245	3.680 ± 0.020	0.720 ± 0.042
T_4_	85:15:0	Freeze	15.730 ± 0.270	13.630 ± 0.108	3.60 ± 0.019	0.680 ± 0.063
T_5_	80:20:0	Freeze	15.620 ± 0.427	13.520 ± 0.178	3.530 ± 0.005	0.630 ± 0.085
T_6_	90:10:0	Cabinet	13.810 ± 0.357	12.610 ± 0.178	3.520 ± 0.006	0.510 ± 0.170
T_7_	85:15:0	Cabinet	13.690 ± 0.273	12.530 ± 0.267	3.490 ± 0.009	0.490 ± 0.093
T_8_	80:20:0	Cabinet	12.840 ± 0.403	12.460 ± 0.278	3.370 ± 0.010	0.460 ± 0.105
T_9_	90:0:10	Freeze	15.960 ± 0.395	13.920 ± 0.195	3.710 ± 0.020	0.730 ± 0.087
T_10_	85:0:15	Freeze	15.750 ± 0.275	13.640 ± 0.088	3.640 ± 0.015	0.690 ± 0.128
T_11_	80:0:20	Freeze	15.807 ± 0.340	13.670 ± 0.118	3.760 ± 0.005	0.700 ± 0.020
T_12_	90:0:10	Cabinet	13.930 ± 0.248	12.670 ± 0.203	3.550 ± 0.007	0.520 ± 0.022
T_13_	85:0:15	Cabinet	13.810 ± 0.428	12.580 ± 0.230	3.540 ± 0.014	0.510 ± 0.042
T_14_	80:0:20	Cabinet	12.890 ± 0.335	12.490 ± 0.160	3.500 ± 0.005	0.530 ± 0.060

*MD, maltodextrin; SPI, soy protein isolate*.

#### Total Flavonoid Content

The data in Table 5 depict that, with an increase in the concentration of carrier agents, the TFC decreased from 13.74 to 12.46 mg RE/g for MD-treated powder and from 13.92 to 12.49 mg RE/g for SPI-treated powder in both freeze-dried and cabinet dried BMPs, respectively, which was less than that of the untreated powder having 16.08 and 17.31 mg RE/g for the cabinet dried and freeze-dried powders, respectively. Similar results were also reported by Ahmed et al. (54), stating that the lowest flavonoid content was observed in purple sweet potato powders treated with an increased concentration of MD. In contrast, flavonoid content was high in powders treated with lower MD concentration. Muzaffar et al. (57) also observed a similar trend with the TFC in TPP of 5.96 CE mg/100 g, which was lower than fresh ripe tamarind pulp (12.22, 5.96 CE mg/100 g flavonoid content). TFC is highly susceptible to heating and oxidation (26). The TFC of the sample was highly affected by both the concentration of carrier agents and the drying method. More flavonoid retention was attained with freeze-drying than with cabinet drying; this change can be attributed to the temperature variation in the two different drying techniques.

#### DPPH Scavenging Activity

The data present in Table 5 show that, as the concentration of carrier agents increased, the scavenging activity of BMP decreased from 3.68 to 3.37 mg/ml in MD-treated powder and from 3.76 to 3.50 mg/ml in SPI-treated powder in both cabinet dried and freeze-dried BMPs, respectively, which was less than that of the untreated powder having 3.98 and 4.06 mg/ml for the cabinet dried and freeze-dried powders, respectively. In the DPPH assay, the freeze-dried powders achieved better antioxidant activity than cabinet dried samples, which may be because the higher temperature of cabinet drying led to the significant damage of thermolabile antioxidant compounds responsible for hydrogen or electron donors to the DPPH free radical, such as flavonoids, and similar results were reported by Miron et al. (63). Muzaffar and Kumar (61) also found similar results stating that an increase in SPI concentration significantly reduced the DPPH radical scavenging activity of the TPP samples from 61.91 to 45.81%, which can be related to the concentration effect of SPI at each concentration level. The scavenging activity of tamarind pulp can be related to polyphenols and flavonoids contributing to electron transfer or hydrogen donating ability.

#### Reducing Power Activity Assay

The EC_50_ value of the extract represents the concentration depicting 0.5 absorbances. The data present in Table 5 show that, with the incorporation of carrier agents, the reducing power decreased from 0.72 to 0.46 mg/ml in MD-treated powder and from 0.73 to 0.51 mg/ml in SPI-treated powder in both cabinet dried and freeze-dried BMPs, respectively, which was less than that of the untreated powder having 0.76 and 0.83 mg/ml for cabinet dried and freeze-dried powders, respectively. Silva et al. (64) also obtained similar results, stating that the reducing capacity was higher for powders treated with 15% MD and 30% gum arabic and lower for powders treated with 30% MD. The reduction in the reducing capacity of powders prepared with carrier agents was mainly due to component dilution (64). Variations in the values of bioactive components in samples can be due to the different carrier agents and different drying methods.

The influence of drying conditions on the antioxidant activity of the dried products was investigated by examining the relationship between TPC, TFC, DPPH and reducing power activity of the samples obtained under various drying conditions. Table 6 shows the Pearson correlation between TPC, TFC, DPPH, and the reducing power activity of the samples obtained under various drying conditions. TPC and TFC were shown to have a significant (*p* < 0.05) and positive association with antioxidant activity under different drying conditions.

**Table 6:** Pearson's correlation coefficient for total phenolic content (TPC), total flavonoid content (TFC), 2,2-diphenyl-1-picrylhydrazyl (DPPH), and reducing power activity of BMP.

**Correlated components**	**TPC (mgGAE/g)**	**TFC (mg RE/g)**	**DPPH radical scavenging activity (IC_**50**_; mg/ml)**	**Reducing power activity (EC50; mg/ml)**
TPC (mg GAE/g)	1			
TFC (mg RE/g)	0.95087*	1		
DPPH Radical Scavenging Activity (IC50; mg/ml)	0.92247*	0.86424*	1	
Reducing power activity (EC50; mg/ml)	0.91525*	0.95036*	0.8853*	1

Two-tailed test of significance is used. *Correlation is significant at the 0.05 level.

### FTIR Analysis

**Figure 5:**
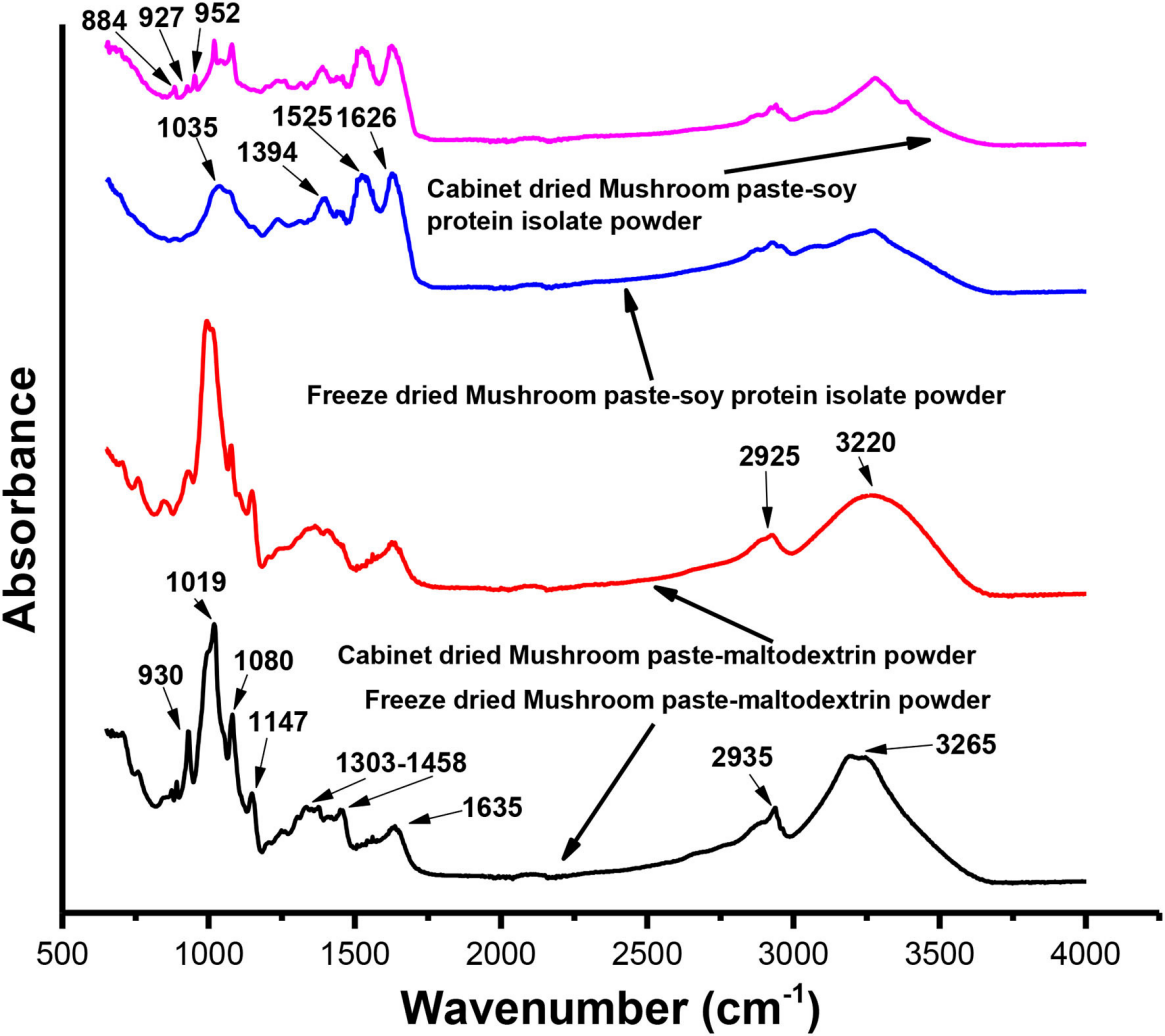
Fourier transform infrared (FTIR) analysis of freeze-dried and cabinet dried mushroom powders.

Fourier transform IR analysis of the formulated powder products was carried out in the wavenumber range 650–4,000 cm^−1^ to study the chemical bond structures and identify the functional groups of the products. The IR spectrum of the developed products is shown in Figure 5. The freeze-dried powder product of the mushroom paste–MD showed significant peaks at 930, 1,019, 1,080, 1,147, 1,303–1,458, 1,635, 2,935, and 3,220 cm^−1^. The cabinet dried powder product of the mushroom paste–MD showed characteristic peaks at 930, 994, 1,077, 1,148, 1,305–1,458, 1,628, 2,925, and 3,265 cm^−1^. Major peaks of the cabinet dried powder product of the mushroom paste–SPI were observed at 1,035, 1,394, 1,525, 1,626, 2,927, and 3,271 cm^−1^. For the freeze-dried mushroom paste–SPI, significant peaks were observed at 884, 927, 952, 1,019, 1,078, 1,388, 1,524, 1,625, 2,939, and 3,277 cm^−1^.

The wavenumber absorption band in the range of 800–1,300 cm^−1^ has been considered the “carbohydrate region” and is characterized by sharp overlapping peaks resulting from glycosidic linkages consisting of CO stretching, CC stretching, and COH bending vibrations (65–70). The wavenumber absorption peak in the range of 1,303–1,358 cm^−1^ could be ascribed to the N–H bending and C–N stretching of the “amide III group” of tertiary aromatic amines. The peak wavenumber in the range of 1,625–1,635 cm^−1^ could be C=O stretching of the “amide I group” of the antiparallel β-pleated sheet protein structures present in mushrooms (70–79). A characteristic absorption peak in the wavenumber range of 2,927–2,939 cm^−1^ could be assigned due to the CH stretching and denotes the presence of carbohydrates. The peak at 3,188–3,277 cm^−1^ could be assigned to the O–H stretching vibrations or N–H stretching groups (70, 71, 73, 80–82).

The reduction in the peak intensity of the freeze-dried mushroom–MD sample at wavenumbers of 930 and 1,077 cm^−1^ and a slight absorption peak shift from 1,080 to 1,077 cm^−1^ were observed. The reduction of peak intensities and the absorption peak shift from higher to lower wavenumbers indicates that the freeze-dried powder product has a weaker bonding structure than the cabinet-dried powder product. Deformation and weakening of the bonding structure could make the powder product less stable, resulting in a shortened shelf life. Absorption peaks of the mushroom–SPI powder at wavenumbers of 1,524–1,525 cm^−1^ could be assigned to the amide II region in proteins (83, 84). This bonding type of protein might be from the SPI (which is also very nutritious due to its high protein content) component of the powder. The amide II band at 1,524–1,525 cm^−1^ is very stable under different conditions (85). The N–H groups and O–H in the SPI component could form intra- and interhydrogen bonding with the C=O moiety of the protein structure (amino acids) of SPI and the mushroom. The formation of the –C–H bond was confirmed by the appearance of additional peaks at wavenumbers of 884, 927, and 952 cm^−1^ in cabinet dried mushroom paste and SPI powder, confirming the formation of undesirable different complex compounds. The formation of different complex compounds was undesirable for our target powder product. Hence, due to the presence of SPI without the formation of additional complex compounds, the freeze-dried powder product of the mushroom paste incorporated with SPI could be considered the best product because it is relatively more stable in addition to its higher protein content compared to others.

## Conclusion

The present study investigated the performance of different carrier agents, such as MD and SPI, and button mushrooms during both freeze-drying and cabinet drying. The influence of incorporation of carrier agents at different concentrations on functional, flow, bioactive, and chemical parameters was investigated. SPI showed a positive increase in powder recovery. MD showed better solubility, and SPI displayed higher hygroscopicity. The results of this study also confirmed the efficacy of the addition of carrier agents to retain bioactive compounds. The highest recovery of phenolic compounds, calculated using FTIR of treated samples, depicted that the incorporation of carrier agents was proven to be effective, and freeze-drying was the most appropriate drying technique. It was concluded that SPI had higher potential as a carrier agent to produce BMPs during drying when compared with MD. BMPs are high in bioactive components that have antioxidant characteristics, such as phenolic and flavonoid compounds. Mushroom powders can be used as a supplement for a variety of staple foods because of their capacity to boost protein content and also to provide valuable health benefits from bioactive compounds.

## Data Availability Statement

The original contributions presented in the study are included in the article/supplementary material, further inquiries can be directed to the corresponding author/s.

## Author Contributions

RS, JS, KD, and AD contributed to the conception, design of the study, and wrote the first draft of this manuscript. GN and AD proposed the title of this manuscript. RS, JS, and KD wrote the sections of this manuscript. BK, AS, AA, HH, and MA have critically revised this manuscript. All authors collated papers, read, and approved the final version of this manuscript.

## Conflict of Interest

The authors declare that the research was conducted in the absence of any commercial or financial relationships that could be construed as a potential conflict of interest.

## Publisher's Note

All claims expressed in this article are solely those of the authors and do not necessarily represent those of their affiliated organizations, or those of the publisher, the editors and the reviewers. Any product that may be evaluated in this article, or claim that may be made by its manufacturer, is not guaranteed or endorsed by the publisher.
